# Dual-targeting by CRISPR/Cas9 leads to efficient point mutagenesis but only rare targeted deletions in the rice genome

**DOI:** 10.1007/s13205-019-1690-z

**Published:** 2019-03-28

**Authors:** Bhuvan Pathak, Shan Zhao, Muthusamy Manoharan, Vibha Srivastava

**Affiliations:** 1 Department of Crop, Soil and Environmental Sciences, University of Arkansas, Fayetteville, AR 72701 USA; 2 Cell and Molecular Biology Program, University of Arkansas, Fayetteville, AR 72701 USA; 3 Department of Agriculture, University of Arkansas at Pine Bluff, Pine Bluff, AR 71601 USA; 4 Department of Horticulture, University of Arkansas, Fayetteville, AR 72701 USA

**Keywords:** CRISPR/Cas9, Multiplex gene editing, Genomic deletion, Point mutations, Genome engineering

## Abstract

The present study investigated the efficiency of CRISPR/Cas9 in creating genomic deletions as the basis of its application in removing selection marker genes or the intergenic regions. Three loci, representing a transgene and two rice genes, were targeted at two sites each, in separate experiments, and the deletion of the defined fragments was investigated by PCR and sequencing. Genomic deletions were found at a low rate among the transformed callus lines that could be isolated, cultured, and regenerated into plants harboring the deletion. However, randomly regenerated plants showed mixed genomic effects, and generally did not harbor heritable genomic deletions. To determine whether point mutations occurred at each targeted site, a total of 114 plants consisting of primary transgenic lines and their progeny were analyzed. Ninety-three plants showed targeting, 60 of which were targeted at both sites. The presence of point mutations at both sites was correlated with the guide RNA efficiency. In summary, genomic deletions through dual-targeting by the paired-guide RNAs were generally observed in callus, while de novo point mutations at one or both sites occurred at high rates in transgenic plants and their progeny, generating a variety of insertion–deletions or single-nucleotide variations. In this study, point mutations were exceedingly favored over genomic deletions; therefore, for the recovery of plant lines harboring targeted deletions, identifying early transformed clones harboring the deletions, and isolating them for plant regeneration is recommended.

## Introduction

Genome-editing effects are based on the creation of double-stranded breaks (DSB) in the target DNA that are repaired by the cell through non-homologous end-joining (NHEJ) or homology-directed repair (HDR) pathways (Jasin and Haber 2016; Waterworth et al. 2011). While HDR leads to predictable outcomes as determined by the DNA template, NHEJ ends up with insertions, deletions and/or substitutions (Puchta et al. 1996; Rouet et al. 1994; Szostak et al. 1983), leading to gene knockouts. The power of CRISPR/Cas9 lies in its efficiency in creating DSBs in genomic sequences containing NGG protospacer adjacent motif (PAM). The simplified version of CRISPR/Cas9 consists of a single-guide (sg) RNA bound to Cas9 (sgRNA:Cas9) that targets genomic sequences through RNA–DNA pairing. Although, sgRNA design is based on a relatively simple 5′-N(20)-NGG-3′ targeting rule (Cong et al. 2013; Jinek et al. 2012; Mali et al. 2013; Mojica et al. 2009), the efficiency of different sgRNAs could vary in the cell. Therefore, multiple sgRNAs are often used in creating targeted knockouts. As a result, targeted genomic deletions by CRISPR/Cas9 have been observed in numerous studies.

Dual-targeting by CRISPR/Cas9, based on the paired use of sgRNAs, could generate somatic and heritable deletions of genomic fragments. Short deletions of ~ 100 bp are frequently reported in plants (Brooks et al. 2014; Kapusi et al. 2017; Nekrasov et al. 2017; Ordon et al. 2017). Dual-targeting was also effective in deleting larger fragments (~ 0.5 kb, ~ 0.7 kb, and 1.6 kb) as reported in maize, kiwi fruit, and rice (Minkenberg et al. 2017; Shi et al. 2017; Srivastava et al. 2017; Wang et al. 2018). Fragments of 10–12 kb could be deleted in rice and Arabidopsis (Durr et al. 2018; Wang et al. 2017a), and even larger fragments of 170–245 kb were deleted by multiplex targeting in rice (Zhou et al. 2014). The efficiencies of genomic deletions varied greatly in these reports, but short deletions (~ 100 bp) were obtained more readily than large deletions (Kapusi et al. 2017; Ordon et al. 2017; Wang et al. 2017a). However, compared to point mutagenesis (effect of a single sgRNA), genomic deletions (effect of paired sgRNAs) consistently occurred at much lower rate even when two or more sgRNAs of equal efficiencies were used (Minkenberg et al. 2017; Tian et al. 2017; Wang et al. 2017a, b).

The application of CRISPR/Cas9 in genome editing is limited by the DNA repair pathways of the host organism. In somatic cells of plants and other higher organisms, NHEJ is the major repair pathway (Puchta et al. 1996; Waterworth et al. 2011); therefore, targeted mutagenesis is the most successful application of CRISPR/Cas9. Another genomic effect that could be created by NHEJ is fragment deletion by a pair of sgRNAs to simultaneously create DSBs at two different sites on a segment of the genome (dual-simultaneous targeting). Ligations of the two distal ends through NHEJ would effectively delete the intervening fragment. Genomic deletions could serve as useful editing effects in functional genomics and biotechnology by targeting gene clusters, *cis*-regulatory elements or transgenes. However, current understanding of dual-targeting by CRISPR/Cas9 in creating genomic deletions is narrow. Many studies have reported genomic deletions, but little is known about the efficiency and success in recovering stable plants lines harboring the defined deletion.

The present study investigated the efficiency of obtaining defined genomic deletions of 240 bp, 945 bp, and 1637 bp from three different loci by dual-targeting in rice. Defined deletions were detected by PCR among transformed calli, and as expected, plants regenerated from these calli harbored the deletions and transmitted to their progeny. However, randomly regenerated plants harboring mixed genomic effects either did not show deletions or showed a low rate of somatic deletions. Furthermore, while targeting frequency of each sgRNA increased in the progeny, genomic deletions remained undetectable. Therefore, for ensuring the recovery of plant lines harboring deletions defined by dual-targeting, it is recommended to screen early transgenic clones (calli) and isolate the characterized clones for plant regeneration. The recovery of de novo deletion lines through plant screening and progeny analysis, at least in rice, appears to be highly unlikely.

## Results

### Experimental design

**Fig. 1:**
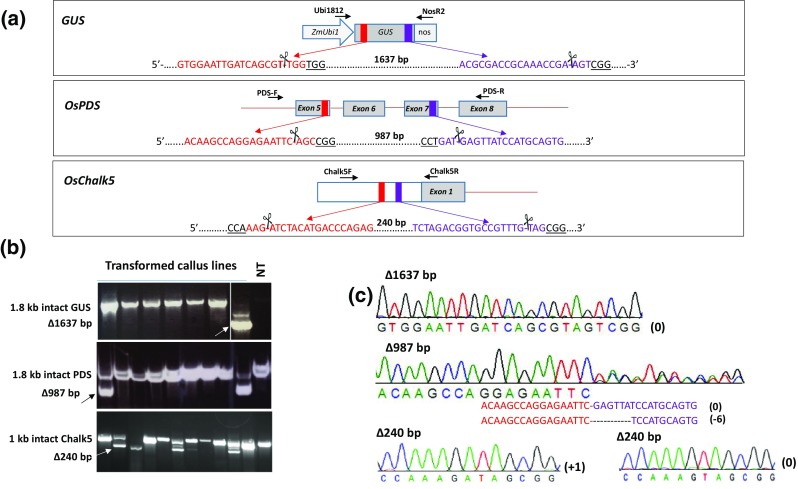
Dual-targeting by CRISPR/Cas9 for fragment deletions. **a** Paired sgRNAs for targeting three genes, transgene *GUS* and native genes*, OsPDS* and Os*Chalk5,* in rice. Full structure of *GUS* gene and partial structures of *OsPDS* and *OsChalk5* genes are shown with sgRNA (red and purple boxes) and primer (arrows) locations. sgRNA spacer 1 (red) or sgRNA spacer 2 (purple) for each locus are shown with protospacer adjacent motif (PAM) (underlined). The positions of double-stranded break (DSB) sites are shown by scissors that defined deletion sizes given in base pairs (bp). *ZmUbi* refers to maize Ubiquitin-1 promoter and *nos* to nopaline synthase 3′ transcription terminator. *GUS* and *OsPDS* genes are targeted in the genic regions (exons), while *OsChalk5* in the intergenic region, upstream of promoter harboring *cis*-elements (white box). **b** PCR screening of callus clones using forward and reverse primers spanning targeted sites (see Table 1; **a**). Representative callus lines are shown with non-transgenic controls (NT; cv. Nipponbare). The intact and the deletion fragments (∆) are indicated; **c** Sequences of the representative deletion fragments of *GUS* (∆1637 bp), *PDS* (∆987 bp), and *Chalk5* (∆240 bp) loci. The number of bases representing insertion–deletions (indels) is given in parentheses

The efficiency of CRISPR/Cas9 in deleting genomic fragments was estimated on three loci, *GUS* transgene (AF485783), rice *PDS* (LOC_Os03g08570), and rice *Chalk5* (LOC_Os05g06480.1; Chromosome 5: 3,335,405–3,341,600) (Fig. 1a). Two sites in each locus were chosen based on *5′*-N(20)-NGG-*3′* rule (Cong et al. 2013; Jinek et al. 2012; Mali et al. 2013), with the goal of creating deletions through simultaneous targeting by a pair of sgRNAs (sg1 + sg2). While *GUS* and *PDS* sgRNAs targeted the genic regions, *Chalk5* sgRNAs targeted an intergenic region harboring *cis*-regulatory elements (Fig. 1a). To generate sg1 and sg2 from a single vector, oligonucleotides containing sgRNA spacers were cloned in pRGE32, which contains tRNA splicing mechanism to generate multiple sgRNAs from a single transcript produced by the rice *U3* promoter (Xie et al. 2015). The resulting *GUS*-, *PDS*- or *Chalk5*- targeting vectors, pJU24, pJU34, and pJU46, respectively, were transformed into the B1 rice line, expressing the *GUS* gene, or the wild-type Nipponbare rice. Line B1 that contains a single-copy of *GUS* gene has been described earlier (Nandy and Srivastava 2012). The resulting transgenic lines were screened by PCR to identify deletions in *GUS*, *PDS,* or *Chalk5* genes, indicated by amplification of fragments shorter by 1637 bp, 987 bp, and 240 bp, respectively (Fig. 1a). A representative PCR indicating genomic deletion in the three loci is shown in Fig. 1b. Targeted deletion of *GUS* in the callus lines has been described earlier (Srivastava et al. 2017). This work further investigated genomic deletions on two more loci, *PDS* and *Chalk5*, and analyzed plant lines to determine the rates of genomic deletions and point mutations through amplicon sequencing by the Sanger method.

### Detection of genomic deletions in callus lines

**Fig. 2:**
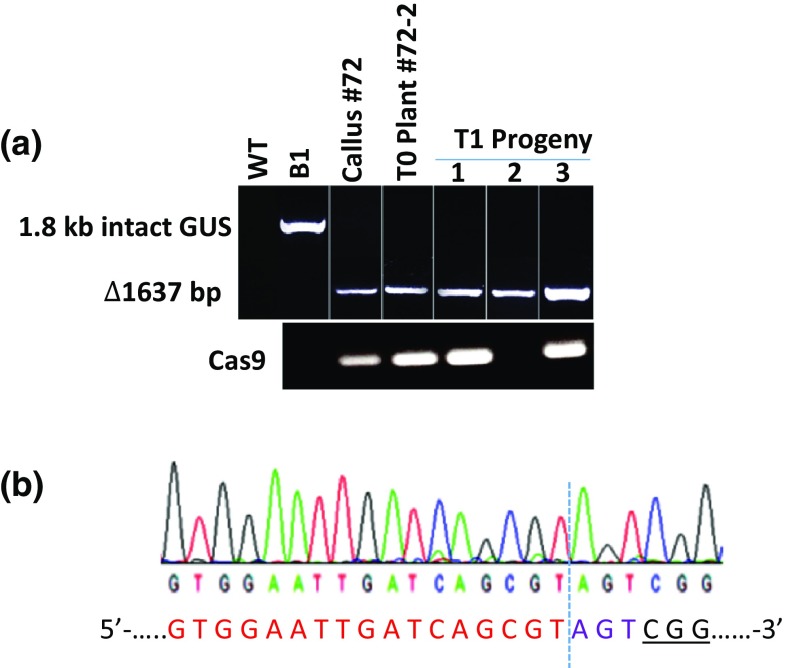
Recovery of stable plant lines harboring ∆1637 bp *GUS* deletion. **a** PCR analysis to detect *GUS* and Cas9 in the callus, primary transgenic plant (T0), and the progeny (T1). WT, wild-type Nipponbare; B1, transgenic *GUS* line; **b** DNA sequencing spectrum of ∆1637 bp fragment in T0 plant#72-2 generated by the paired used of sgRNAs. The observed sequence matches the predicted deletion site derived from joining of distal ends without indels. Dashed vertical line indicates blunt DSB ligation

Genomic deletions (Δ) in the callus lines transformed with pJU24, pJU34, or pJU46 were tested by PCR and indicated by the respective Δ amplicons observed in a PCR (Fig. 1b). As reported earlier, *GUS* deletion in pJU24-transformed lines occurred in 2 out of 113 callus lines (Srivastava et al. 2017). In the present study, genomic deletions in two additional loci, *PDS* and *Chalk5* loci, were determined in pJU34- and pJU46-transformed lines (Table 1). Genomic deletions at *PDS* locus was found in 2 out of 32 callus lines and at *Chalk5* locus in 4 out of 53 callus lines. Sequencing of the Δ amplicons indicated that the distal ends, created by the blunt DSBs, ligated without indels or with short indels to generate the Δ locus. The indels generally consisted of insertion or deletion of a single nucleotide or a few nucleotides (Fig. 1c), which is consistent with other studies that report single-nucleotide variations as most common outcome of CRISPR/Cas9 targeting (Mao et al. 2013, van Overbeek et al. 2016). One of the pJU46 lines (*Chalk5*) showed an amplicon ~ 0.2 kb larger than the intact *Chalk5* amplicon. Sequencing of this amplicon showed insertion of 0.2 kb fragment of unknown source in one of the targeted sites (single-site targeting, data not shown). Overall, the efficiency of creating genomic deletions by dual-targeting was low and variable with the sgRNA pairs (sg1 + sg2). Targeted deletions by *GUS* sgRNA pairs were reported in only 1.7% of the transformed callus lines (Srivastava et al. 2017). The *PDS* and *Chalk5* sgRNA pairs, on the other hand, generated significantly higher rates of deletion at somewhat similar rates in the callus lines (Table 1). Nevertheless, these observations indicate that genomic deletions could be created through dual-targeting by CRISPR/Cas9, and as reported earlier, calli harboring Δ locus could be regenerated into plants (Srivastava et al. 2017). Plants regenerated from one of the callus lines (line#72) contained homozygous Δ locus, indicated by the presence of Δ1637 bp amplicon and absence of 1.8 kb amplicon in the PCR. As expected, the progeny of this plant inherited the stable Δ locus that independently segregated from *Cas9* (Fig. 2a). The sequence of the Δ1637 bp in these plants was consistent with the creation of DSB at the predicted sites (3-bp upstream of PAM in each targeted site) followed by ligation of the distal ends without indels (Fig. 2b).

**Table 1:** Genomic deletion by dual-targeting in callus lines

Exp.	Target Gene	Vector	Predicted Δ size (bp)	Total lines	PCR detection	DNA sequencing		Eff. (%)^a^
						(−) InDel	(+) InDel	
1	*OsPDS*	pJU34	985	32	2	–	2	6.2
2	*OsChalk5*	pJU46	240	53	4	2	2	7.5

^a^Percent events showing genomic deletion by PCR as shown in Fig. 1. *GUS* deletion data is given in Srivastava et al. 2017

### Targeting efficiency in plants

**Fig. 3:**
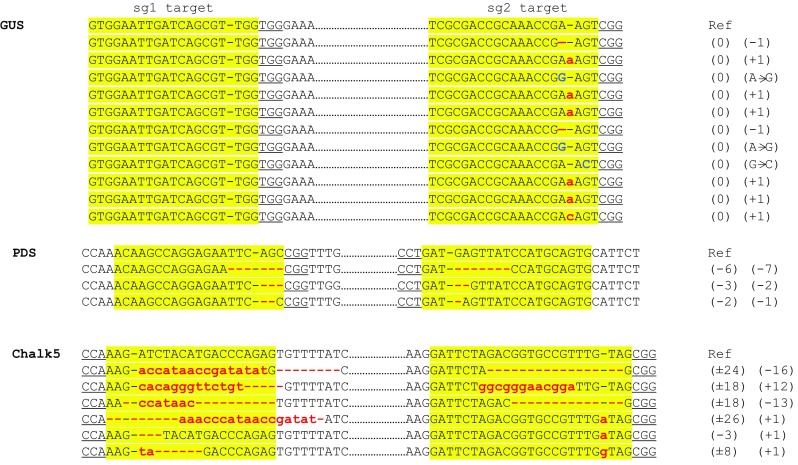
Types of mutations observed in T0 plants. Sequence alignments of *GUS*, *PDS* and *Chalk5* sequences at sg1 and sg2 targeted sites (yellow highlights). PAM sequences are underlined, and DSB site is shown as (−) in each reference sequence. Insertion/deletions/substitutions for each site are shown on the right. Deletions are shown as red dashes, insertions as small red letters, and substitutions as large blue letters

As described above, plant lines carrying the defined Δ locus could be regenerated from calli harboring the deletion. In the same experiment, a number of chimeric T0 plants were also regenerated that showed somatic deletions indicated by the presence of two amplicons, indicative of intact locus and Δ locus, in the same PCR reaction (Srivastava et al. 2017). However, when these chimeric plants were analyzed at a later stage of growth (flowering) in the greenhouse, the Δ1637 bp amplicon was undetectable, in spite of testing multiple tissue from different tillers of each plant. This observation suggests that the young regenerated plants harbored somatic deletions that are unlikely to be transmitted to the progeny. Among *PDS* and *Chalk5* T0 plants, genomic deletions were undetectable by PCR at both early and late stages of growth (data not shown). To investigate the individual effect of each sgRNA, T0 plants were characterized for the presence of point mutations at each targeted site. A total of 50 T0 plants, representing *GUS*, *PDS,* or *Chalk5* targeting were analyzed by PCR and sequencing (Table 2). Some of these GUS plants selected for this analysis showed Δ1637 bp amplicon in the leaf tissue of the young regenerated plants (Srivastava et al. 2017). Twelve of the 21 GUS plants did not show mutations at either targeted sites. The remaining nine showed targeting but only at sg2 target. Of the 12 PDS lines, 3 lacked targeting, while 9 contained targeting at both sites. Finally, 6 out of 17 Chalk5 lines lacked targeting, and the remaining contained targeting at both sites (Table 2). T0 plants were mostly chimeric for targeting, as 2 or more traces were observed in the characteristic superimposed overlapping peaks downstream of the DSB site in the sequencing spectra. Analysis of these traces revealed the types of mutations found at the DSB sites (Fig. 3). In summary, targeting efficiency of the two *GUS* sgRNAs was highly dissimilar, but the two *PDS* or *Chalk5* sgRNAs showed similar targeting efficiency (Table 2). Sequence alignments of the targeted sites revealed interesting observations: (1) the targeted *GUS* site in all 9 T0 plants contained only a single-nucleotide variation consisting of 1 bp insertion, deletion or substitution at the predicted DSB site; (2) the two targeted *PDS* sites contained short deletions ranging from 1 to 7 bp, with only one line containing a larger deletion; and (3) the targeted *Chalk5* sites showed most diverse types of mutations with short indels and 1 bp insertions at the two DSB sites (Fig. 3). These observations suggest that possibly genomic context, target sequence, and sgRNA efficiency influence the outcome of CRISPR/Cas9 targeting. In support, a recent study in yeast showed that types of indels generated by CRISPR/Cas9 depended on DNA sequence context and PAM orientation (Lemos et al. 2018).

**Table 2:** Point mutations in primary transgenic (T0) plants

Exp.	Target	Total no. of plants	Non-targeted	No. of plants targeted^a^		Eff. (%)^b^	
				sg1 site	sg2 site	sg1	sg2
1	*GUS*	21	12	0	9	–	42
2	*OsPDS*	12	3	9	9	75	75
3	*OsChalk5*	17	6	11	11	64	64

^a^Generally chimeric mutations observed. Types of mutations shown in Fig. 3

^b^Percent plants harboring mostly chimeric mutations at predicted DSB sites

### Targeting in progeny plants

**Fig. 4:**
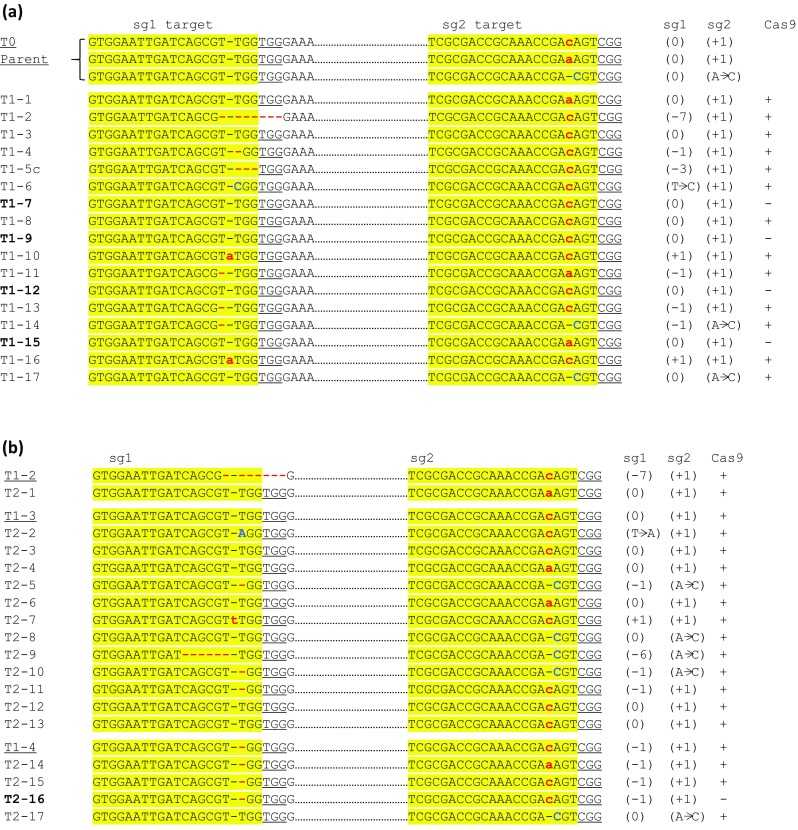
Genotyping of progeny plants derived from the T0 parent expressing GUS-targeting vector. **a** T1 progeny, and **b** T2 progeny. The mutation types in sg1 and sg2 targets are shown, see Fig. 3 for notations. Bold T1/T2 lines are Cas9-negative. Parent plants are underlined with their representative progeny given below

To investigate inheritance of CRISPR-induced deletions, 61 progeny seedlings derived from three GUS T0 plants were analyzed by PCR. None of the progeny, however, showed Δ1637 bp amplicon, indicative of stable genomic deletion. These plants were also stained for GUS activity, 34 of which were negative, indicating targeting at sg1 and/or sg2 sites. To determine the inheritance of point mutations, selected GUS-negative progeny derived from a single parent plant was analyzed and compared with the parent plant that contained chimeric targeting at sg2 site. In the parent plant, no targeting was evident in sg1 site, but three types of mutations were observed at the predicted sg2 DSB site: + 1 (A or C) and A-to-C substitution (Fig. 4a); however, + 1 C was the most commonly observed mutation in multi-sample analysis that likely rendered the plant GUS negative. None of the T1 plants showed Δ1637 bp amplicon; however, de novo targeting by sg1 was frequently observed. Eight of the 17 T1 plants showed chimeric targeting (≥ 2 types of sequences) at sg1 target. The most common type of mutation at sg1 target was 1 bp deletion; however, 1 bp insertion and longer deletions were also observed (Fig. 4a). The analysis of sg2 target among T1 plants revealed that all 17 plants contained mono-allelic or biallelic mutations (Table 3). Biallelic mutations were either identical on each allele (homozygous) or different (heterozygous). The alignment of sequences revealed that all observed mutations were also present in the parent. Four T1 plants (T1–7, 9, 12, 15) had segregated from Cas9 gene, confirming inheritance of the mutation (Fig. 4a). In summary, while targeting at both sites was observed in T1 plants, de novo genomic deletions were undetectable.

**Table 3:** Point mutations in *GUS*-CRISPR/Cas9 progeny

Locus	Generation	No. of plants tested	sg1 mutations^a^				sg2 mutations^a^			
			Non-targeted	Mono-allelic	Bi-allelic^b^	Chimer.^c^	Non-targeted	Mono-allelic	Bi-allelic^b^	Chimer.^c^
GUS	T1	17	9	6	–	2	–	12	5	–
GUS	T2	17	8	7	1	1	–	10	7	–
Chalk5^d^	T1	30	0	0	30	–	7	8	15	–

^a^Types of mutations shown in Figs. 4 and 5

^b^Heterozygous or homozygous

^c^Presence of > 2 overlapping traces downstream of DSB site in the sequencing spectra

^d^T1 plants of *Chalk5* from potentially same transgenic event but different T0 plants

Next, T2 progeny derived from three T1 plants (T1–2, T1–3, and T1–4) were analyzed by PCR and sequencing. Once again, no genomic deletion was detected in any of the T2 plants. The three T1 parents all contained identical mutation at sg2 site (+ 1 C), but differed at sg1 site. T1–2 contained 7 bp deletion at sg1 site, but its progeny completely lacked mutations at sg1 sites and contained de novo single-nucleotide variation (+ 1 A) at sg2 site, indicating that mutations observed in the parent were not heritable and de novo mutations were introduced. T1–3 lacked mutations at sg1 site and contained C insertion at sg2 site. Its T2 progeny showed de novo mutations at sg1 site: single bp variation (insertion/deletion/substitution) and 6 bp deletion, whereas at sg2 site, both inheritances of + 1 C insertion and de novo single-base variations were observed. T1–4 contained − 1 T in sg1 site and + 1 C at sg2 target. Its T2 progeny, one of which lacked Cas9, inherited these mutations; however, new mutations were also observed: + 1 A and A–C substitution (Fig. 4b). All of these mutations were observed in the T1 parents; therefore, mutations at sg2 target were likely inherited, but de novo mutations were also created. Inheritance of mutation was confirmed in one T2 plant that contained − 1 and + 1 at the sg1 and sg2 sites, respectively (Fig. 4b). In summary, while genomic deletions remained undetectable, increased rate of point mutations (effect of single sgRNA) was observed in T1 and T2 progeny with single-base variation as the common type of mutation at the targeted site. We also investigated whether single-base variations frequently found at sg2 site could alone confer *GUS* negative phenotype as observed in T0 parent plant. We found that A–C substitution did not change the protein sequence, but + 1 A and + 1 C generated frame shift and early stop codon (data not shown), mutating the C-terminal catalytic domain of β-glucuronidase (GUS) enzyme (Wallace et al. 2010), leading to inactivation of GUS activity.

**Fig. 5:**
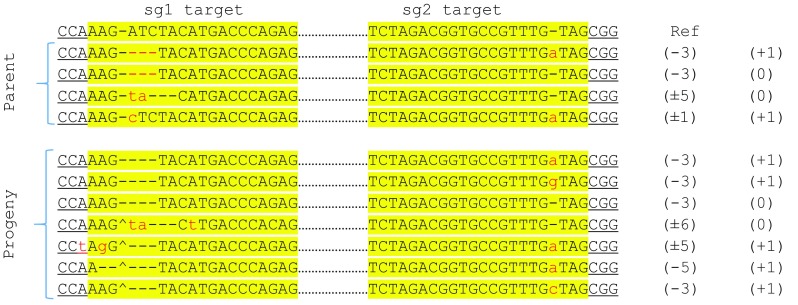
Genotyping of progeny plants derived from the T0 parent expressing Chalk5-targeting vector. The mutation types in sg1 and sg2 targets in the parent and progeny plants are aligned with the reference, see Fig. 3 for notations

We also analyzed T1 progeny of *Chalk5* T0 plants that showed chimeric effects at sg1 and sg2 sites by superimposed overlapping peaks downstream of the DSB site in the sequencing spectra. The analysis of the spectra by CRISP-ID tool identified short deletions at sg1 site and 1 bp insertions (+ 1) at sg2 site (Fig. 5). Thirty T1 plants from this chimeric parent were analyzed by PCR and sequencing. No deletion was evident, but point mutations at each site were found as homozygous or heterozygous mutation (Table 3; Fig. 5). Furthermore, at least one of the mutations identified in the parent plant (− 3 at sg1 and + 1 at sg2) was transmitted to the progeny at high rate.

### Same mutation pattern from different targeting events

**Fig. 6:**
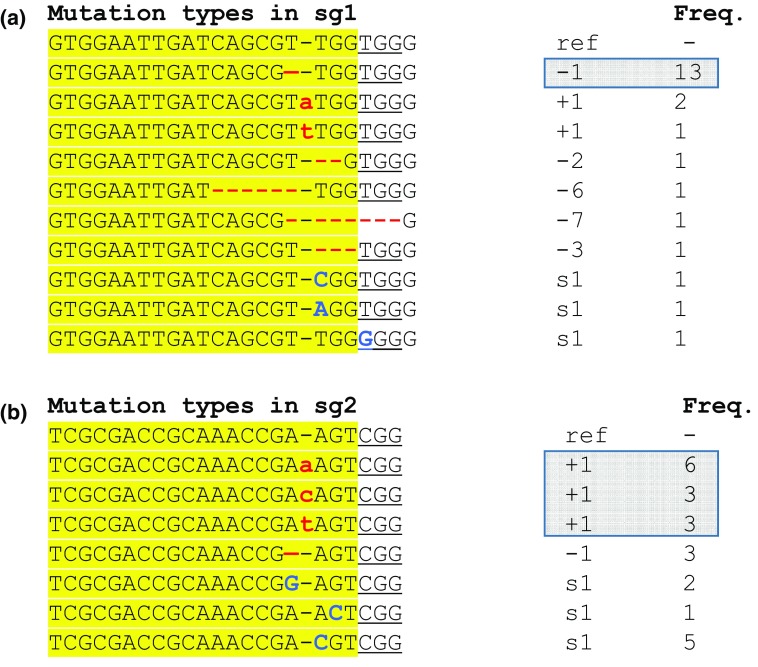
Frequency of mutations observed at *GUS* targets as determined by Sanger sequencing of the sg1 target (**a**) and sg2 target (**b**). The reference sequences with PAM (underlined) and DSB site (−) are shown on the top. Insertions (+) and deletions (−) are shown in red and substitutions (s) in blue fonts. s1 refers to single-nucleotide substitution at or near DSB site. Frequency refers to number of times a mutation type observed among the 23 lines. Boxed numbers indicate most common mutation types (− 1 or + 1) and their frequency

We frequently observed − 1 and/or + 1 mutations at *GUS* sg1 and sg2 sites in the targeted lines. To investigate whether the same type of mutation arises from different targeting events, we compared *GUS* sg1 and sg2 sites in 23 different lines obtained from 3 different experiments. At sg1 site, deletion of a single nucleotide (− 1) at the DSB site was observed 13 times (Fig. 6a), whereas at sg2, insertion of a single nucleotide (+ 1) at the DSB site was observed 12 times (Fig. 6b). The next most frequent type of mutation was single-base substitution (s1), which either occurred at the DSB site or in the PAM (Fig. 6a, b). Other types of mutations at the two sites included short deletions or single-nucleotide variations, which were generally observed once in the population. In summary, the repair of sg1 and sg2 DSB sites led to a predictable mutation pattern of − 1 or + 1 in ~ 50% of the transformed lines generated within the experiment or between experiments.

## Discussion

Plant genome engineering involves a variety of genomic modifications including gene insertion, replacement, inactivation, or deletion. Creating predictable genetic variation is highly desirable, but often defeated by the host repair processes that ignore DNA homologies and generate unpredictable mutations in higher plants (Jasin and Haber 2016; Puchta et al. 1996; Waterworth et al. 2011). As a result, targeted knockout is the most common outcome of genome editing. Genomic deletions, however, do not rely on homology-based DNA repair and, therefore, should be possible to create by standard gene-editing methods.

One of the applications of targeted genomic deletion is transgene excision to rid transgenic plant of antibiotic-resistance marker genes. While effective methods of transgene removal are available, they require specialized vector constructions, e.g., adding recombination sites or separating marker gene from the gene-of-interest in two T-DNAs (Gidoni et al. 2008; Komari et al. 1996; Wang et al. 2011). On the other hand, CRISPR/Cas9 can target loci by virtue of the cloned sgRNA spacers (Cong et al. 2013; Jinek et al. 2012; Mali et al. 2013), thereby, giving more flexibility to the user. Genomic deletion could also be pursued to create null mutations to allow detection by standard PCR, while screening of small indels would require mismatch cleavage assay, DNA sequencing, quantitative, or digital PCR (Belhaj et al. 2013; Falabella et al. 2017; Kim et al. 2009; Voytas 2013; Xie and Yang 2013). Genomic deletions could also create useful traits. The natural variant of rice *DEP1* harbors Δ625 bp that confers erect panicles and increased grain yield (Huang et al. 2009), and the spontaneous deletions in maize *WAXY* gene alter starch composition of the grains (Wessler et al. 1990). Genomic deletions also play major roles in plant evolution (De Smet et al. 2017; Soltis et al. 2014). Divergence in the function of the duplicated genes could occur upon deletions in the genes (Haberer et al. 2004; Liu et al. 2011). For example, deletions in the intergenic regions could either remove or change the position of *cis*-elements leading to altered tissue specificity and neo-functionalization of the gene (Arsovski et al. 2015; De Smet and Van de Peer 2012). Thus, targeted genomic deletions could serve as useful effects in plant genome engineering.

CRISPR/Cas9 has emerged as the dominant gene-editing tool that holds a great promise for genome engineering in plants and animals. This study evaluated the practical application of CRISPR/Cas9 in creating targeted genomic deletions in three loci in the rice genome. Previously, we reported successful deletion of *GUS* gene through dual-targeting by CRISPR/Cas9, which was accomplished by PCR screening and regeneration of the selected clones (Srivastava et al. 2017). Zhou et al. (2014) also reported chromosomal deletions in rice calli that were subjected to regeneration to recover plant lines. Similarly, in the present study, dual-targeting was successful in creating genomic deletion in transformed callus lines that mostly correlated with the efficiency of the sgRNA pairs. However, genomic deletions were rarely detected among plants transformed with Cas9:sgRNA constructs, and recovery of stable deletion lines was unsuccessful unless they were derived from calli harboring the deletion. This is somewhat surprising as point mutations by each sgRNA employed in dual-targeting occurred at high frequency, and the efficiency of the two sgRNAs used on two rice loci (*PDS* and *Chalk5*) was comparable. Furthermore, rate of point mutations in the two sites increased dramatically in the progeny, yet targeted deletions remained undetectable. Consistent with our study, others have also reported a much lower rate of genomic deletions by multiplex sgRNAs that is generally one order of magnitude lower than targeted point mutagenesis at two or more sites in the segment of the genome (Durr et al. 2018; Ordon et al. 2017).

At the outset, these observations suggest that multiplex targeting by CRISPR/Cas9 occurs through non-concurrent activity on different sites as a result of dissimilar sgRNA efficiencies. Low rate of deletions in *GUS*, as observed in this study, could be based on dissimilar sg1 and sg2 efficiencies. However, genomic deletions in *PDS* and *Chalk5* that were targeted by equally efficient sgRNA pairs were not proportionately increased. Therefore, understanding of the kinetics of Cas9-generated DSB could lend a mechanistic explanation. The Cas9:sgRNA complex stays bound to the broken termini of the DNA (Jiang and Doudna 2017; Sternberg et al. 2014), which may prevent the free-fragment from being physically removed from the site. Subsequently, the free-fragment could participate in the NHEJ process and eventually be glued back to the genome. Thus, simultaneous DSBs end up with point mutations at each site rather than fragment deletion. Our dual-targeting data on three loci with highly variable efficiencies of sgRNA suggest that although sgRNA efficiency and Cas9 expression are important for the success of targeting, above a threshold, these parameters are unlikely to improve the rate of genomic deletions. Furthermore, DNA repair mechanisms in plants could affect the targeting outcome and enforce DSB repair by preserving broken termini and introducing only small indels, the most commonly observed effect of CRISPR/Cas9 targeting in plants (Mao et al. 2013). Nevertheless, heritability of genomic deletions and other editing effects could be improved by expressing Cas9 by germline promoters (Durr et al. 2018; Feng et al. 2018). Finally, the survey of mutations in multiple transformed lines obtained from different experiments showed that the same type of mutation occurred frequently in the DSB sites. While sg1 site mostly lost a nucleotide (− 1), the sg2 site gained one (+ 1). The mechanistic explanation of this curious observation is not clear, but it implicates the role of target site and/or genomic context. More analysis with additional sgRNAs is needed to better understand the frequency of a given type of mutation in CRISPR/Cas9 targeting; however, similar observations have been made by Jacobs et al. (2015), who found identical mutation in multiple soybean lines. In a separate study based on targeting 10 loci in rice, + 1 was found to be the most common mutation (> 50%), followed by − 1 (Zhang et al. 2014). However, our data suggest that a target site could also have the preference for either an insertion (+ 1) or a deletion (− 1).

In summary, consistent with a previous report on CRISPR/Cas9 targeting in rice (Jang et al. 2016), this study found that primary regenerated plants mostly harbor chimeric mutational effects. However, since the observed effects are generally not heritable, PCR screening at an early stage of callus growth, and isolation of the calli harboring the deletions will be an important step in recovering stable deletion lines. In addition, this study found that the types of mutations induced at a specific site by CRISPR/Cas9 are not highly variable, and frequently, the same type of mutation is observed from different targeting events. This observation suggests that DSB repair is highly dependent on the target sequence.

## Materials and methods

### DNA constructs and plant transformation

The sgRNA spacer sequences were selected using CRISPR RGEN tool (http://www.rgenome.net/cas-designer/; Park et al. 2015). Vector pRGE32 (Addgene#63159) was used for synthesizing the CRISPR/Cas9-targeting vectors pJU24, pJU34, and pJU46 against *GUS* (NCBI accession no. AF485783), *OsPDS* (Os03g08570), and *Chalk5* (Chromosome 5: 3,335,405–3,341,600) genes, respectively. The two sgRNAs targeting each gene were expressed as polycistronic tRNA–gRNA (PTG) genes, which was synthesized against pGTR (Addgene# 63143) using the protocol of Xie et al. (2015). The constructed PTG (tRNA–gRNA1–tRNA–gRNA2) was ligated to pRGE32 vector by FokI/BsaI digestions, and the resulting vectors were used for rice transformations. The gRNA oligos used for PTG construction are given in Table 4. For targeting *GUS*, B1 transgenic line (cv. Nipponbare) was used for transformation as described earlier by Srivastava et al. (2017), while Nipponbare was used for targeting rice genes, *OsPDS* and *OsChalk5*. The embryogenic callus from mature seeds was used for all transformations by the gene gun (PDS1000, Bio-Rad Inc.), in which pJU24, pJU34, or pJU46 DNA was co-bombarded with hygromycin phospho-transferase expressing vector, p35S:HPT. The transformed calli were isolated and regenerated on hygromycin (50 mg/l) containing media using the protocol of Nishimura et al. (2006).

**Table 4:** Primers used in the study

Primer	Sequence (5′–3′)	Application
gGus1F	TAGGTCTCCTGATCAGCGTTGGgttttagagctagaa	Construction of sgRNA1 *(GUS*): 5′-GTGGAATTGATCAGCGTTGG-3′
gGus1R	CGGGTCTCAATCAATTCCACtgcaccagccggg	
gGus2F	TAGGTCTCCCCGCAAACCGAAGTgttttagagctagaa	Construction of sgRNA2 (*GUS*): 5′-ACGCGACCGCAAACCGAAGT-3′
gGus2R	CGGGTCTCAGCGGTCGCGTtgcaccagccggg	
gPDS1F	TAGGTCTCCCAGGAGAATTCAGCgttttagagctagaa	Construction of sgRNA1 (*OsPDS*): 5′-ACAAGCCAGGAGAATTCAGC-3′
gPDS1R	CGGGTCTCACCTGGCTTGTtgcaccagccggg	
gPDS2F	TAGGTCTCCATGGATAACTCATCgttttagagctagaa	Construction of sgRNA2 (*OsPDS*): 5′-CACTGCATGGATAACTCATC-3′
gPDS2R	CGGGTCTCACCATGCAGTGtgcaccagccggg	
gChalk1F	TAGGTCTCCTCATGTAGATCTTgttttagagctagaa	Construction of sgRNA1 (*OsChalk5*): 5′- CTCTGGGTCATGTAGATCTT-3′
gChalk1R	CGGGTCTCAATGACCCAGAGtgcaccagccggg	
gChalk2F	TAGGTCTCCGACGGTGCCGTTTGTAGgttttagagctagaa	Construction of sgRNA2 (*OsChalk5*): 5′-GATTCTAGACGGTGCCGTTTGTAG-3′
gChalk2R	CGGGTCTCACGTCTAGAATCtgcaccagccggg	
Ubi1812	TCTAACCTTGAGTACCTATCTATTA	Forward primer in B1 locus
NosR2	GCGGGACTCTAATCATAAAAACCC	Reverse primer in B1 locus
PDSF	GGTAGAAATGCCATGCGGGA	Forward primer in *OsPDS*
PDSR	GTGGTGAGGTTCGGCTGAAT	Reverse primer in *OsPDS*
Chalk5F	ACAAGGCTAGCAAGTTGGC	Forward primer in *OsChalk5*
Chalk5R	CACTCGCTCGTCTTCTCCTC	Reverse primer in *OsChalk5*
Cas9F	AAAGACCGAGGTGCAGACAG	Forward primer in Cas9
Cas9R	ACCAGCACAGAATAGGCCAC	Reverse primer in Cas9

### Molecular analysis

Genomic DNA isolated from callus, regenerated plants or seedlings, was used for polymerase chain reaction (PCR) using primers spanning the target sites (Table 4). The PCR products were resolved on agarose gel and extracted using Geneclean Spin Kit (MP Biomedicals, CA, USA) for sequencing from both ends using forward and reverse primers by the Sanger Sequencing method at Eurofins Genomics USA. The sequences were viewed on Sequence Scanner 2 software (Applied Biosystems Inc.) and aligned with the reference sequences using CLUSTAL-Omega multiple sequence alignment tool. CRISPR-ID tool was used to separate superimposed overlapping spectrum in Sanger sequencing traces, characteristic of heterozygous or chimeric mutations (Dehairs et al. 2016). The type of indel was identified by cloning PCR amplicon into pCR2.1 vector using TA cloning kit (Thermo-Fisher Scientific, NY) as per manufacturer’s instructions and sequencing individual colonies by Sanger sequencing.
